# Lanthanum Oxide Nickel Hydroxide Composite Triangle Nanosheets for Energy Density Asymmetric Supercapacitors

**DOI:** 10.3389/fchem.2021.783942

**Published:** 2021-11-11

**Authors:** Huiyu Duan, Mei Shi, Mengfei Zhang, Geyu Feng, Suli Liu, Changyun Chen

**Affiliations:** Key Laboratory of Advanced Functional Materials of Nanjing, School of Environmental Science, Nanjing Xiaozhuang University, Nanjing, China

**Keywords:** asymmetric supercapacitor, electrochemical energy storage, nanosheet, transition metal hydroxides, rare earth

## Abstract

Transition metal hydroxides are a kind of promising electrode material in electrochemical energy storage, but the poor conductivity limits their application. Lanthanides are good proton conductors and can usually improve the intrinsic conductivity of other materials. By integrating the merits of lanthanide elements and transition metal hydroxide, we designed lanthanum oxide nickel hydroxide composites (LONH) with unique ultrathin triangle nanosheet morphology via a controllable synthetic strategy for high-performance supercapacitors. When the LONH is used as positive electrode material in aqueous asymmetric supercapacitor, it reveals an energy density (107.8 W h kg^−1^ at 800 W kg^−1^), rate performance (86.9% retention at 4 kW kg^−1^) and outstanding cycle stability (more than 90% retention after 3,000 cycles). This work confirms that compositing La_2_O_3_ and Ni(OH)_2_ can significantly improve the supercapacitor performance of both pristine La_2_O_3_ and transition metal hydroxide composites. We hope this work would offer a good prospect for developing other lanthanide-transition metal hydroxide composites as an attractive class of electrode materials in electrochemical energy storage.

## Introduction

Transition metal hydroxides (TMHs) and derivatives have attracted much attention as electrode materials for application in electrocatalysis (Gong et al., 2013; Fan et al., 2014; Han et al., 2016; Sun et al., 2018; Liu et al., 2019; Zhang et al., 2021a; Zhang et al., 2021b), electrochemical analysis (Tan et al., 2017), and especially electrochemical energy storage (Chen et al., 2014; Zuo et al., 2016; Yao et al., 2018; Jiang et al., 2019; Zhang et al., 2019, 2019; Wang et al., 2020; Yin et al., 2020, 2020; Gürbüz et al., 2021; Jiang et al., 2021; Song et al., 2021b; Song et al., 2021a; Zhang et al., 2021a). As a critical member of TMHs, nickel hydroxides show great potential for high-performance electrode with much lower cost than novel metal-based materials. Thus, Ni-based hydroxides have become a popular electrode material for electrochemistry (Song et al., 2021b). In addition, Ni-based hydroxides usually possess good Faradic activity, which means they are suitable for electrochemical energy storage. There are many meaningful works about Ni-based hydroxides applied for electrochemical energy storage. For example, Zhang et al. reported Co(CO_3_)_0.5_(OH)/Ni_2_(CO_3_)(OH)_2_ nanobelts as positive electrode materials for flexible asymmetric supercapacitor, displaying a high energy density of 22.7 Wh kg^−1^ at a power density of 24,019 W kg^−1^ (Zhang et al., 2020). Chen et al. reported single crystalline *β*-Ni(OH)_2_ quasi-nanocubes used for aqueous Ni-Zn batteries, exhibiting high areal energy and power density (Chen et al., 2021). However, the poor electrical and ionic conductivity of Ni-based hydroxides restricts their many applications in electrochemistry. Thermal treatment can convert hydroxides to oxides, which is a good approach to improve the conductivity, but this process is usually accompanied by layer collapse and structural change, leading to the attenuation of capacitance.

Lanthanide compounds have been widely utilized in many fields (e.g., photocatalysis, photoelectrocatalysis, bioimage) because of the singular optical properties (Liu et al., 2011; Zhou et al., 2015; Escudero-Escribano et al., 2016; Han et al., 2017; Hosseinpour-Mashkani and Sobhani-Nasab, 2017; Regmi et al., 2017; Li et al., 2019). Additionally, most lanthanide compounds are good proton conductors, which can enhance the ion and electron conductivity of electrode materials composited in it (Duan et al., 2019; Ghosh et al., 2019; Duan et al., 2020; Rabani et al., 2021). In our previous works, we found that the addition of lanthanide elements can significantly influence morphology, thickness, and electrochemical properties. In one case, Yb was introduced in Ni(OH)_2_ to obtain Ni_4_Yb(OH)_10_NO_3_·3H_2_O hexagonal nanosheets (Zhu et al., 2021). After addition of Yb, there was significant reduction in thickness of Ni(OH)_2_ nanosheets. When used as electrode material for supercapacitor, the nanosheets showed high capacity that was over 3 times of pristine Ni(OH)_2_. Because it is easier for Yb^3+^ to devote charges to a conductive carrier from the shell 4f^13^ of the Yb^3+^ path and because of reduction in thickness, the electron and ion transport routes were both shortened, resulting in better electrical and ionic conductivity. La, as the representative element of lanthanides, possesses most of the typical properties of lanthanide elements. When added into transition metal-based materials, it can enhance the electrochemical performance. For example, Chakrabarty et al. by La doping enhanced the electrochemical performance of Ni(OH)2/carbon nanotube hybrid electrodes. Despite the fact that the hybrid electrodes show high capacitance (2,731 F g^−1^ at 1 A g^−1^), the energy density (∼25 Wh kg^−1^ at ∼1 kW kg^−1^) is unsatisfactory because of the low charge voltage.

In our study of lanthanide elements and Ni-based hydroxide composites, we found that the addition of lanthanum can change the morphology of Ni(OH)_2_ to ultrathin triangle nanosheets and also significantly boost the supercapacitor performance. In this work, a facile method was used to synthesize lanthanum oxide nickel hydroxide composites (LONH) triangle nanosheets, and they were used as electrode materials for supercapacitors. The LONH triangle nanosheets present an outstanding specific capacitance (783.0 F g^−1^ at 1 A g^−1^), which is about double that of pristine Ni(OH)_2_. Moreover, an aqueous asymmetric supercapacitor (ASC) was assembled with LONH, exhibiting a very high energy density, up to 107.8 W h kg^−1^ at 800 W kg^−1^. This synthetic strategy can also expand to the reliable production of other lanthanide–TMH composites, and we hope it can boost the improvement of electrochemical energy storage.

## Results and Discussion

**FIGURE 1:**
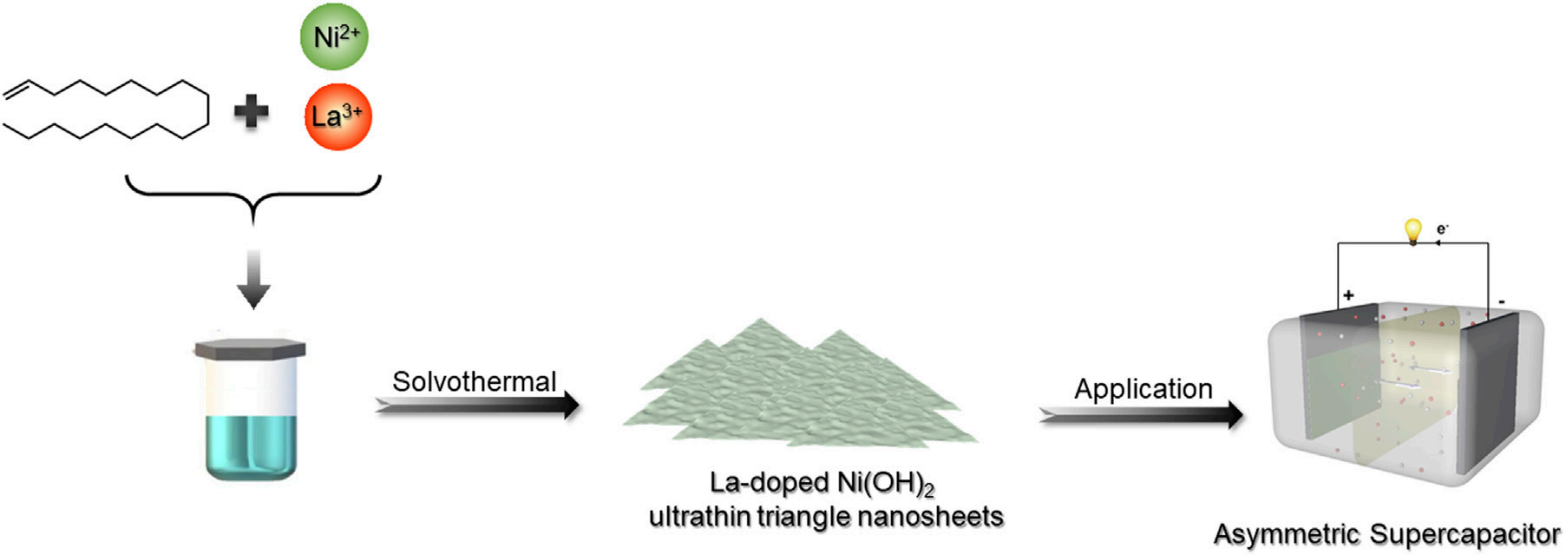
A schematic illustration of the formation of LONH ultrathin triangle nanosheets.

**FIGURE 2:**
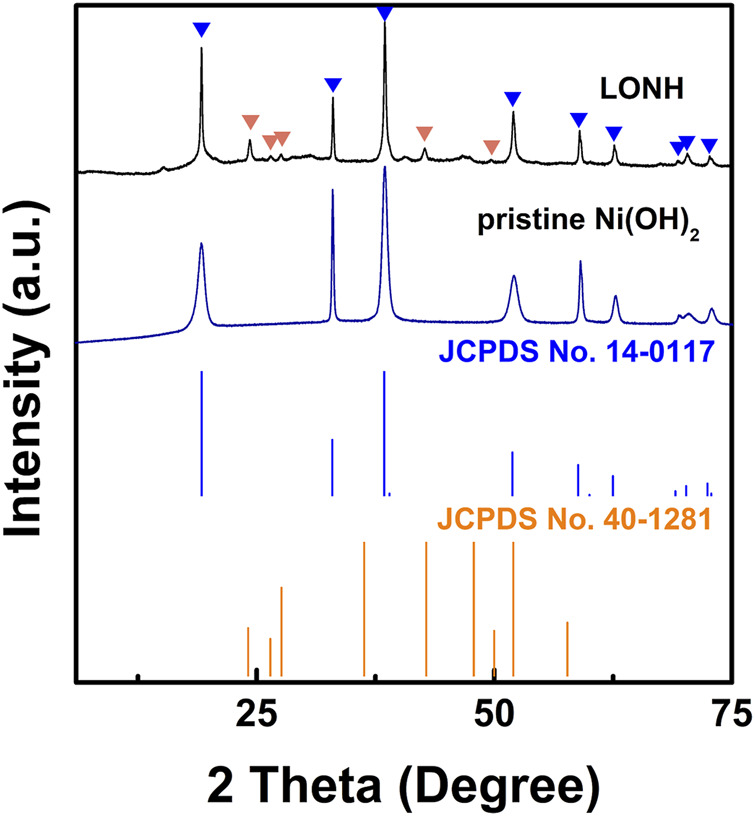
XRD patterns of the as-prepared LONH, Ni(OH)_2_ samples, as well as Ni(OH)_2_ (JCPDS No. 14-0117) and La_2_O_3_ (JCPDS No.40-1281). The peaks from Ni(OH)_2_ and La_2_O_3_ are signified by blue and orange triangles, respectively.

The fabrication of LONH is schematically shown in Figure 1 via a facile method (Supporting Information shows the more synthetic details). X-ray diffraction (XRD) characterized the structure of LONH (Figure 2). The pattern demonstrates that the LONH consists of Ni(OH)_2_ (JCPDS No. 14-0117) and La_2_O_3_ (JCPDS No.40-1281). There are eight peaks (signed by blue triangles) corresponding to the (001), (100), (101), (102), (110), (111), (103), and (201) facets of Ni(OH)_2_, respectively. Four peaks (signified by orange triangles) correspond to the (100), (002), (101), and (110) facets of La_2_O_3_.

**FIGURE 3:**
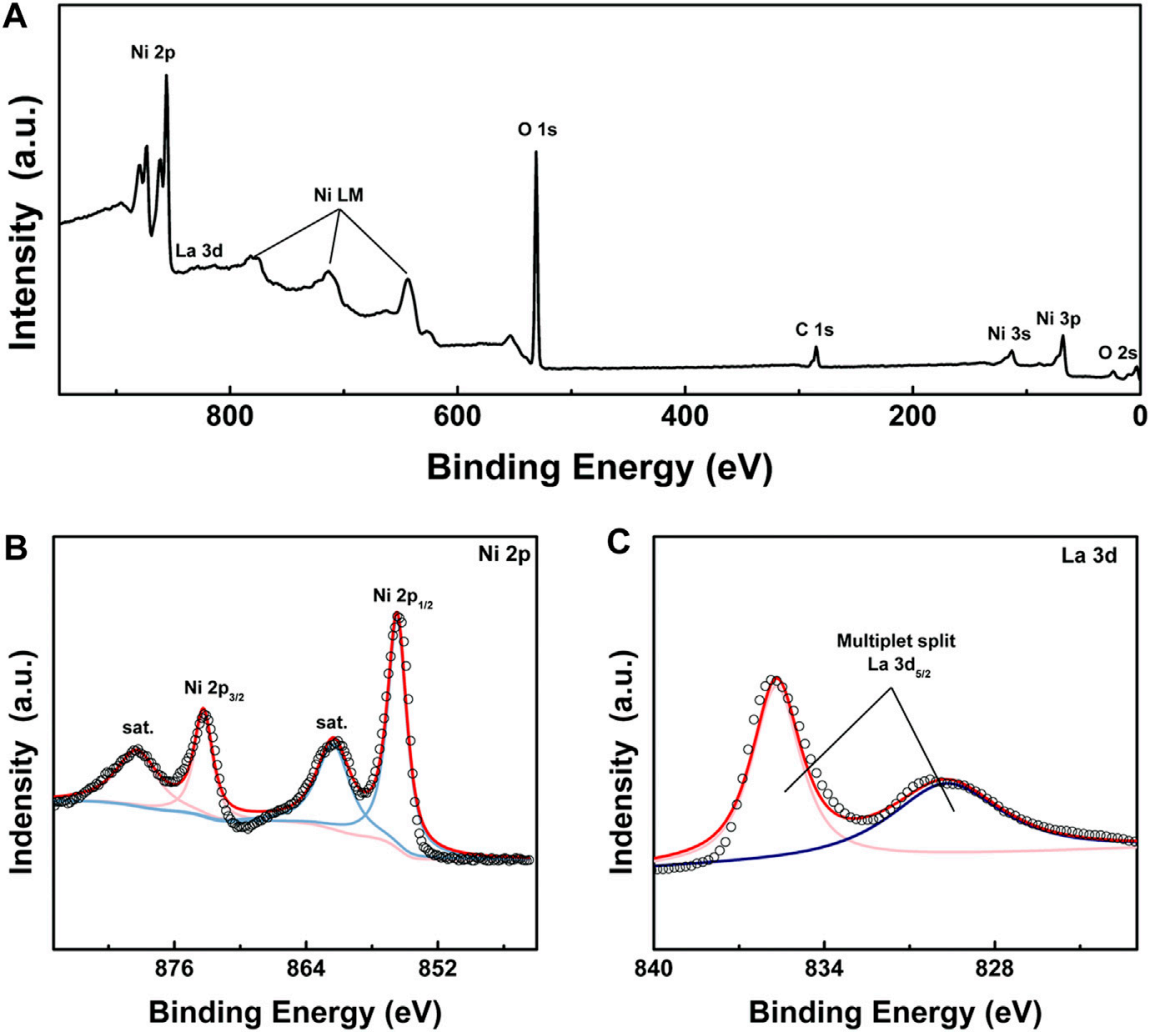
**(A)** XPS spectrum of LONH; **(B)** high-resolution XPS spectra of Ni 2p in LONH; **(C)** high-resolution XPS spectra of La 3d in LONH.

The element composition and chemical state of LONH were examined by X-ray photoelectron spectroscopy (XPS). Figure 3A presents the survey spectrum, demonstrating that Ni, La, O, C, and a little N are evident. Figure 3B is the Ni 2p high-resolution spectrum, revealing two peaks from Ni 2p_1/2_ and Ni 2p_3/2_ signals of Ni(II) at 855.7 and 873.2 eV accompanying the satellite peaks at 861.3 and 879.4 eV. Figure 3C shows the two peaks at 830.5 and 835.2 eV, in accordance with multiplet split La 3d_5/2_. The ΔE of multiplet split La 3d_5/2_ is 4.9 eV, which indicates that the existence form of La is La_2_O_3_, corresponding to the result of XRD analysis.

**FIGURE 4:**
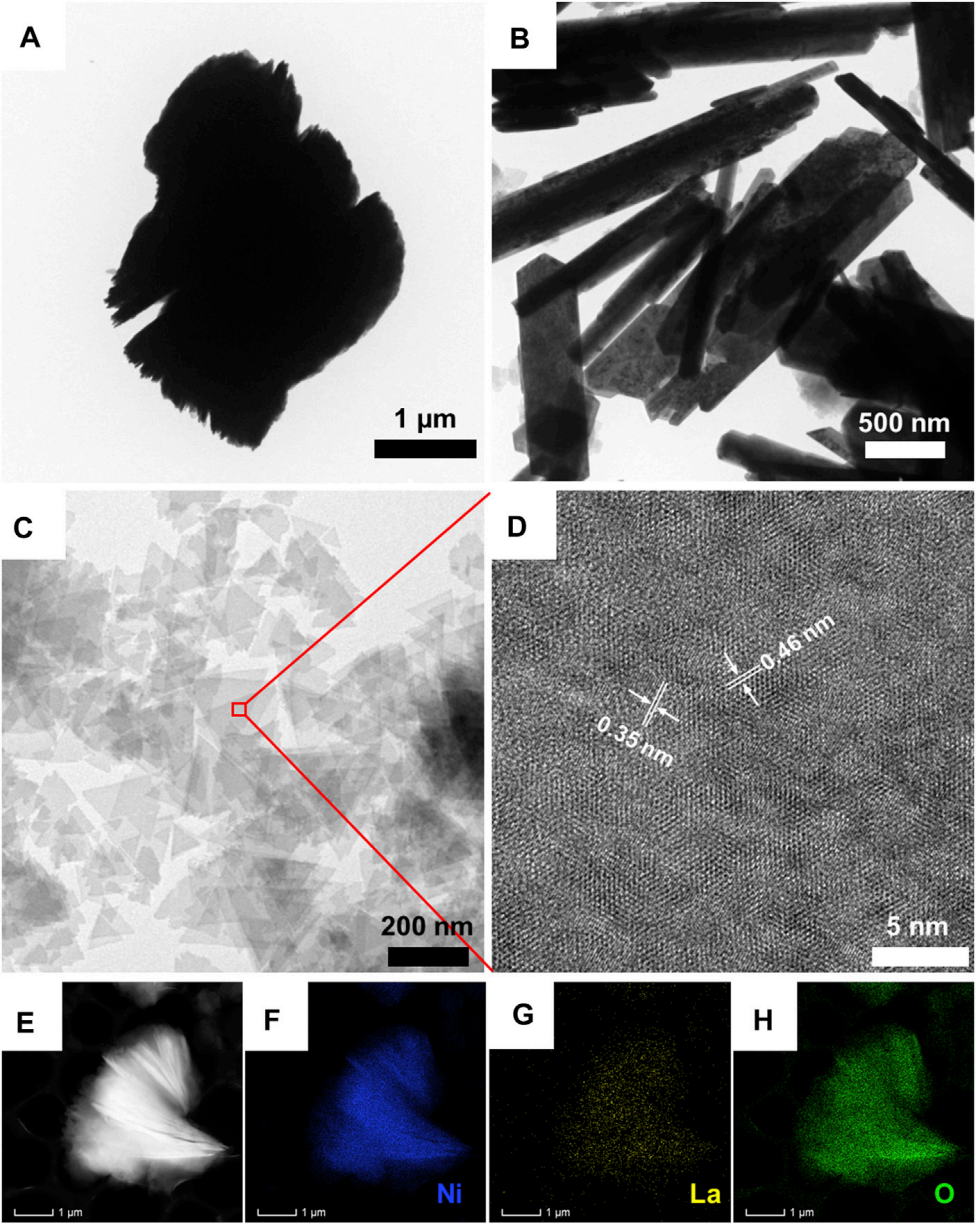
TEM images of **(A)** pristine Ni(OH)_2_, **(B)** pristine La_2_O_3_, and **(C)** LONH; **(D)** HRTEM image of LONH; **(E–H)** energy dispersive spectroscopy (EDS) mapping images of LONH.

Transmission electron microscopy (TEM) was used to disclose the morphological features of pristine Ni(OH)_2_, La_2_O_3_, and LONH. According to Figure 4A, pristine Ni(OH)_2_ possesses a heavily aggregated multiple layer structure. Figure 4B shows the morphology of pristine La_2_O_3_, demonstrating the nanorod-like structure. Interestingly, once La is added into Ni(OH)_2_ in the form of La_2_O_3_, the morphology of Ni(OH)_2_ will significantly change. As shown in Figure 4C, unlike pristine Ni(OH)_2_ and La_2_O_3_, LONH consists of unique dispersed ultrathin triangle nanosheets. High-resolution transmission electron microscopy (HRTEM) was used to further study the phase composition of LONH in Figure 4D. There are two main interplanar spacings of 0.35 and 0.46 nm, corresponding to (100) facet of La_2_O_3_ and (001) facet of Ni(OH)_2_, respectively, and in good agreement with the main peaks of La_2_O_3_ and Ni(OH)_2_ in the XRD pattern. Figures 4E–H show the element mapping images of La, Ni, and O in LONH, indicating that La, Ni, and O are uniformly distributed in LONH nanosheets.

Cyclic voltammetry (CV) is a necessary method in electrochemistry to figure out the redox behavior of LONH. Supplementary Figure S1 shows the CV curves at different scan rates from 10 to 50 mV s^−1^ in electrolyte of 3 M KOH. The CV curves indicate that the redox behavior of LONH mainly consists of Ni(OH)_2_, with the existence of OH^−^. The large area enclosed by CV curves indicates the outstanding Faradic process, which results in an excellent pseudocapacitive performance. The integrate method was used to calculate the capacity of LONH from CV curves, indicating that the specific capacities of LONH are 889.4, 881.0, 852.0, 813.1, and 780.7 F g^−1^ at 1.0–5.0 A g^−1^.

$$\text{Ni}{\left(\text{OH}\right)}_{2}+{\text{OH}}^{-}=\text{NiOOH}+{\text{H}}_{2}\text{O}+e^{-}$$

The galvanostatic charge–discharge (GCD) was used to investigate the specific capacitance of LONH. Supplementary Figure S2 shows the GCD curves within a voltage window of 0.15–0.55 V at different current densities of 1.0–5.0 A g^−1^, in which the platforms confirm the presence of redox processes, corresponding to the CV results. In line with the GCD curves, the specific capacitance of LONH was calculated to be 783.0, 717.5, 659.3, 631.8, and 616.3 F g^−1^ at current densities of 1.0–5.0 A g^−1^, respectively. Supplementary Figure S3 exhibits the capacitances of LONH in different current densities to investigate the rate performance (78.7% retention at 5.0 A g^−1^). To compare the capacitance of LONH and pristine Ni(OH)_2_, their GCD curves are shown in Supplementary Figure S4, revealing that the capacitance of LONH is about double that of pristine Ni(OH)_2_. It demonstrates that the addition of La can obviously enhance the energy storage performance of pristine Ni(OH)_2_.

**FIGURE 5:**
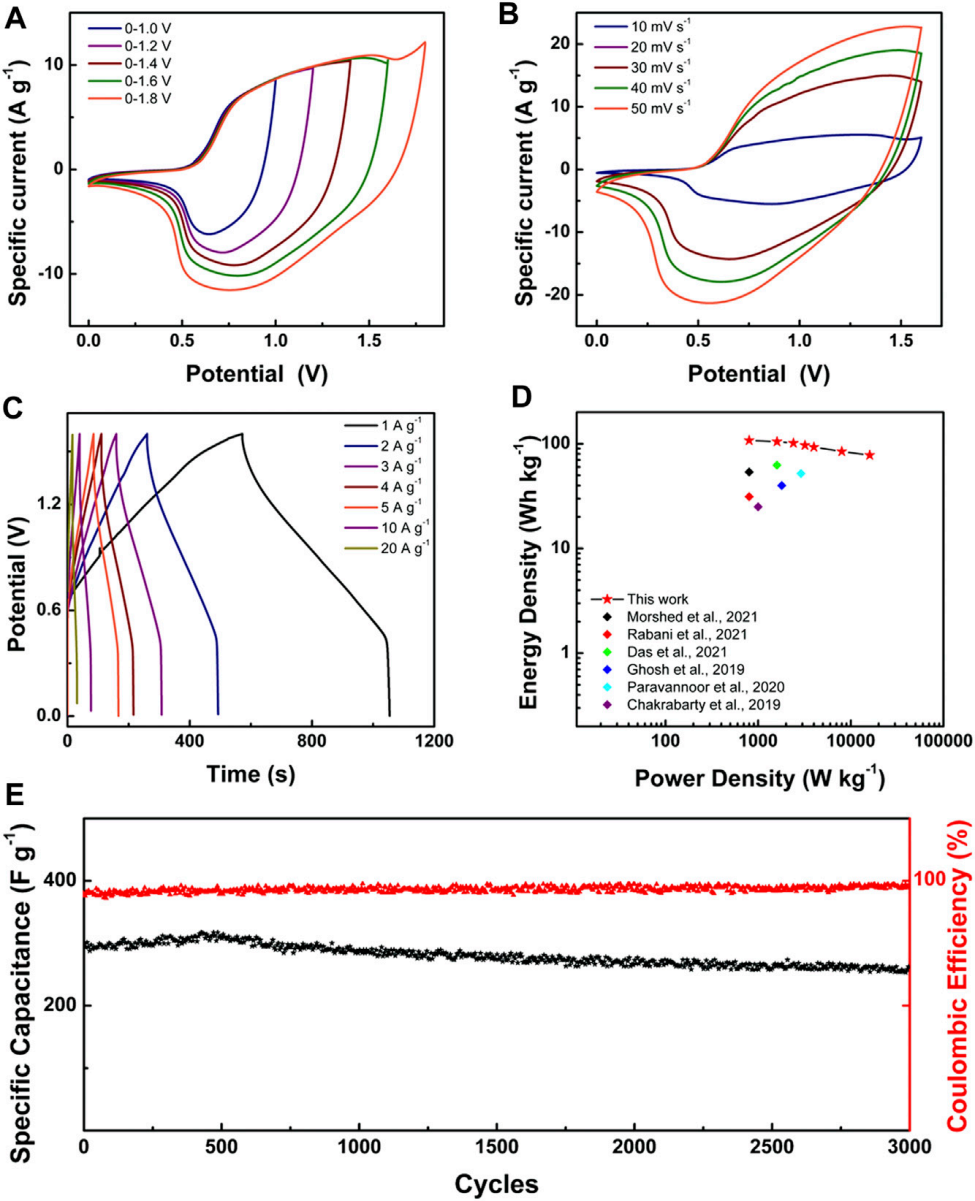
**(A)** CV curves at scan rate of 20 mV s^−1^ of LONH//AC at voltage windows of 0–1.0 to 0–1.8 V in 3.0 M KOH; **(B)** CV curves at scan rates of 10–50 mV s^−1^ of LONH//AC at a voltage window of 0–1.6 V; **(C)** GCD curves of LONH//AC with current densities of 1.0–20.0 A g^−1^; **(D)** Ragone plots of LONH//AC and other reported asymmetric supercapacitors; **(E)** specific capacitance and Coulombic efficiency of LONH//AC during charge–discharge cycling test at 2.0 A g^−1^.

For practice application, we assembled an aqueous ASC device in which the positive electrode used LONH and the negative electrode used activated carbon (AC), respectively (denoted as LONH//AC). The CV (Supplementary Figure S5) and GCD (Supplementary Figure S6) measurements were applied on AC, revealing that the specific capacity of AC is 86.3 F g^−1^ at 1.0 A g^−1^. Because the specific capacity of LONH is 783.0 F g^−1^ at 1.0 A g^−1^, to balance the charge during charging and discharging, the ratio of LONH and AC is about 1:8.8. In order to figure out the suitable voltage window of LONH//AC, CV analysis results with different voltage windows of 0–1.0, 0–1.2, 0–1.4, 0–1.6, and 0–1.8 V are shown in Figure 5A. When the charging voltage is above 1.6 V, obvious electrolyte decomposition occurs, indicating that the most suitable voltage window is 0–1.6 V. The CV and GCD curves of LONH//AC at divergent scan rates were shown in Figures 5B,C. The LONH//AC reaches an energy density of 107.8 W h kg^−1^ at 800 W kg^−1^ and exhibits outstanding rate performance of 86.9% retention at 4 kW kg^−1^. Even at a power density of 16 kW kg^−1^, it still remains 77.8 W h kg^−1^, benefited from the shortened electron and ion transport routes in LONH nanosheets. According to the above test results, LONH//AC is significantly better than previously reported ASCs (Supplementary Table S1), such as PANI/La-10 (56.1 W h kg^−1^ at 400 W kg^−1^) (Morshed et al., 2021), Ce-MOF-0.5//AC (31.3 W h kg^−1^ at 800 W kg^−1^) (Rabani et al., 2021), NiV-LDH (2:2)//Bi_2_O_3_ (65.5 W h kg^−1^ at 1,595.2 W kg^−1^) (Das et al., 2021), MOF-Ce (40 W h kg^−1^ at 1,800 W kg^−1^) (Ghosh et al., 2019), PrO_x_/CNT//V_2_O_5_/graphene (52.1 W h kg^−1^ at 2,900 W kg^−1^) (Paravannoor et al., 2020), and La-Ni(OH)_2_/MWCNT (25 W h kg^−1^ at 1,000 W kg^−1^) (Chakrabarty and Chakraborty, 2019). Figure 5D shows the corresponding comparison Ragone plot.

It is critical for these devices to work reliably and stably for a long time. Figure 5E displays the cycling stability result of LONH//AC via continuously charging and discharging at 2.0 A g^−1^. In the 1st cycle, the capacitance of LONH//AC was 294.1 F g^−1^. With cycle number increasing, the capacitance constantly grew and reached the maximum of 317.3 F g^−1^ at about the 400th cycle. After cycling 1,000 times, the capacitance declined to 286.6 F g^−1^. With continuing cycles, the rate decreased. It finally declined to 262.4 F g^−1^ after 3,000 cycles, and still retained about 90% of its initial capacity. The Coulombic efficiency increased from 89.6 to 96.1% during the whole 3,000 cycles, indicating the good reversibility. For comparison, the cycling stability results of Ni(OH)_2_//AC, La_2_O_3_//AC, and LONH//AC are shown in Supplementary Figure S7, which reveals that the capacity retention of Ni(OH)_2_//AC is 63.8% after 2,000 cycles. After ∼300 cycles, the capacity of La_2_O_3_//AC decreased to 0 quickly, demonstrating that the addition of La_2_O_3_ to Ni(OH)_2_ can enhance the capacity and cycle stability.

## Conclusion

In conclusion, lanthanide elements and TMH composites are attractive electrode materials for electrochemical energy storage. In this work, we designed the composite LONH with unique ultrathin triangle nanosheets from lanthanide elements and TMHs. With regard to LONH, not only Ni(OH)_2_ can ensure high pseudo-capacitance arising from the abundant redox active sites but also the addition of La can make Ni(OH)_2_ much more thinner to significantly shorten ion and electron transport pathways. CV and GCD measurements demonstrate that LONH possesses good redox activity and presents an outstanding specific capacity of 783.0 F g^−1^ at 1 A g^−1^. Furthermore, the ASC device (LONH//AC) shows an energy density of 107.8 W h kg^−1^ at a power density of 800 W kg^−1^ and retains 86.9% at 4 kW kg^−1^. According to the result of stability test, LONH//AC shows the capacitance of 262.4 F g^−1^ after 3,000 cycles (90.1% retention of the first cycle) and high Coulombic efficiency of 96.1%. This work infers that the addition of lanthanide oxides to Ni(OH)_2_ can significantly change the morphology and improve the electrochemical energy storage performance of pristine TMHs. We hope this work would provide an inspiration to develop other lanthanide-TMH composites as attractive electrode materials for electrochemical energy storage.

## Data Availability

The original contributions presented in the study are included in the article/Supplementary Material, further inquiries can be directed to the corresponding author.
