# Supplementary material for: Lanthanum Oxide Nickel Hydroxide Composite Triangle Nanosheets for Energy Density Asymmetric Supercapacitors
Source: Front Chem. 2021 Nov 11;9:783942. doi: 10.3389/fchem.2021.783942 (PMC8632525; doi:10.3389/fchem.2021.783942)
Supplement: Supplementary file 1 [file DataSheet1.docx]

**Lanthanum Oxide Nickel Hydroxide Composite Triangle Nanosheets for Energy Density Asymmetric Supercapacitors**

Huiyu Duan^1, 2†^, Mei Shi^1†^, Mengfei Zhang^2^, Geyu Feng^1^, Suli Liu^1^, and Changyun Chen^1*^

^†^These authors have contributed equally to this work and share first authorship

^1^ Key Laboratory of Advanced Functional Materials of Nanjing, School of Environmental Science, Nanjing Xiaozhuang University, Nanjing, 211171, Jiangsu, P. R.China

^2^ School of Chemistry and Chemical Engineering, Institute for Innovative Materials and Energy, Yangzhou University, Yangzhou, 225009, Jiangsu, P. R. China

***Correspondence:**Changyun Chen

cychen@njxzc.edu.cn

Keywords: asymmetric supercapacitor, electrochemical energy storage, nanosheet, transition metal hydroxides, rare earth

### Experimental section

**Synthesis of Ni(OH)_2_ nanosheets**

Ni(OH)_2_ nanosheets were synthesized via a simple solvothermal method. The solvent consists of 1-octadecene, oil acid as well as dodecylamine, and then 0.4976 g nickel (II) acetate tetrahydrate were dissolved in the above solution. After being transferred into a Teflon-lined stainless autoclave which was carefully cleaned in advance, the solution was heated to 453 K at a rate of 3 K min^-1^ and maintained at 453 K for 18 h. Eventually, a mixture of ethanol as well as heptane was used to wash the sample and dried at 323 K over a night.

**Synthesis of La oxides Ni(OH)_2_ composite nanosheets**

La oxides Ni(OH)_2_ composite nanosheets were synthesized via a simple solvothermal method. The solvent consists of 1-octadecene, oil acid as well as dodecylamine, and then 0.4976 g nickel (II) acetate tetrahydrate and 0.7794 g lanthanum (III) nitrate hexahydrate were dissolved in the above solution. After being transferred into a Teflon-lined stainless autoclave which was carefully cleaned in advance, the solution was heated to 453 K at a rate of 3 K min^-1^ and maintained at 453 K for 18 h. Eventually, a mixture of ethanol as well as heptane was used to wash the sample and dried at 323 K over a night. After a typical synthesis, these organic compounds can be recycled by fractional distillation or rotary evaporation.

**Characterization**

The morphological features were characterized by transmission electron microscopy (TEM, JEM-200CX), high-resolution transmission electron microscopy (HRTEM, JEOL-2100F) and energy dispersive energy dispersive X-ray spectrometry (EDS, JSM-5610 LV-Vantage) mapping. X-ray diffraction (XRD) patterns were analyzed on a D/MAX2500VL/PC X-ray diffractometer (Cu Kα radiation: λ= 0.15406 nm). X-ray photoelectron spectroscopy (XPS, PHI 5000 Versa) was used to measure the chemical states, and the spectrometer was equipped with a standard monochromatic Al Kα source (*hv*=1486.6 eV)

**Electrochemical measurement**

All electrochemical measurements were conducted in a typical three-electrode electrochemical cell, which contained a 3 M KOH aqueous solution as the electrolyte. The working electrode (WE) consisted of nickel foam with active materials. The Hg/HgO electrode served as the reference electrode (RE), and the counter electrode (CE) was a Pt electrode. Cyclic voltammetry (CV) measurements were performed on a Metrohm PGSTAT302N electrochemical workstation. The working electrode was made by mixing the as-prepared sample powder, acetylene black, and polytetrafluoroethylene at a weight ratio of 80:15:5, respectively. Then, a piece of nickel foam size of approximately 1 × 1 cm was coated by above powder. Then, the nickel foam was pressed into a thin foil at a pressure of 8 MPa. The mass loading of the active material on the electrodes was about 22 mg. The electrochemical capacitive performance of the electrodes can be measured by cyclic voltammetry (CV), galvanostatic charge/discharge (GDC) curves and stability test. The specific capacity can be calculated from the GCD curves through equations:

$$C_{m}=\frac{I \Delta t}{m \Delta V}$$

**Asymmetric assembly and measurements**

For asymmetric supercapacitor (ASC) device, the as-synthesized Ce-doped Ni-Co Hydroxide nanosheets and activated carbon (AC) used as positive and negative electrodes, respectively, which was investigated in 3 M KOH. The negative electrode was prepared by using a similar procedure with positive electrode. The AC was produced by Shenzhen Naxin Material Co., Ltd whose type is YEC-8. The specific surface area and bulk density of AC(YEC-8) are about 2000 m^2^/g and 0.4 g/mL, respectively. Similar as before, the negative electrode was made by mixing the as-prepared AC, acetylene black, and polytetrafluoroethylene at a weight ratio of 80:15:5, respectively. Then, the above powder was coated on a piece of nickel foam, approximately 1 × 1 cm^2^. Then, the nickel foam was pressed into a thin foil at a pressure of 8 MPa. The mass loading of the active material on the electrodes was about 193.6 mg and the mass ratio of the positive electrode and negative electrode was about 1:8.8 according from the specific capacities of LONH and AC. The energy density (E, W h kg^-1^) and power density (P, W kg^-1^) were calculated through equations:

$$E=\frac{C \Delta V^{2}}{7.2}$$

$$P=\frac{E\times3600}{\Delta t}$$

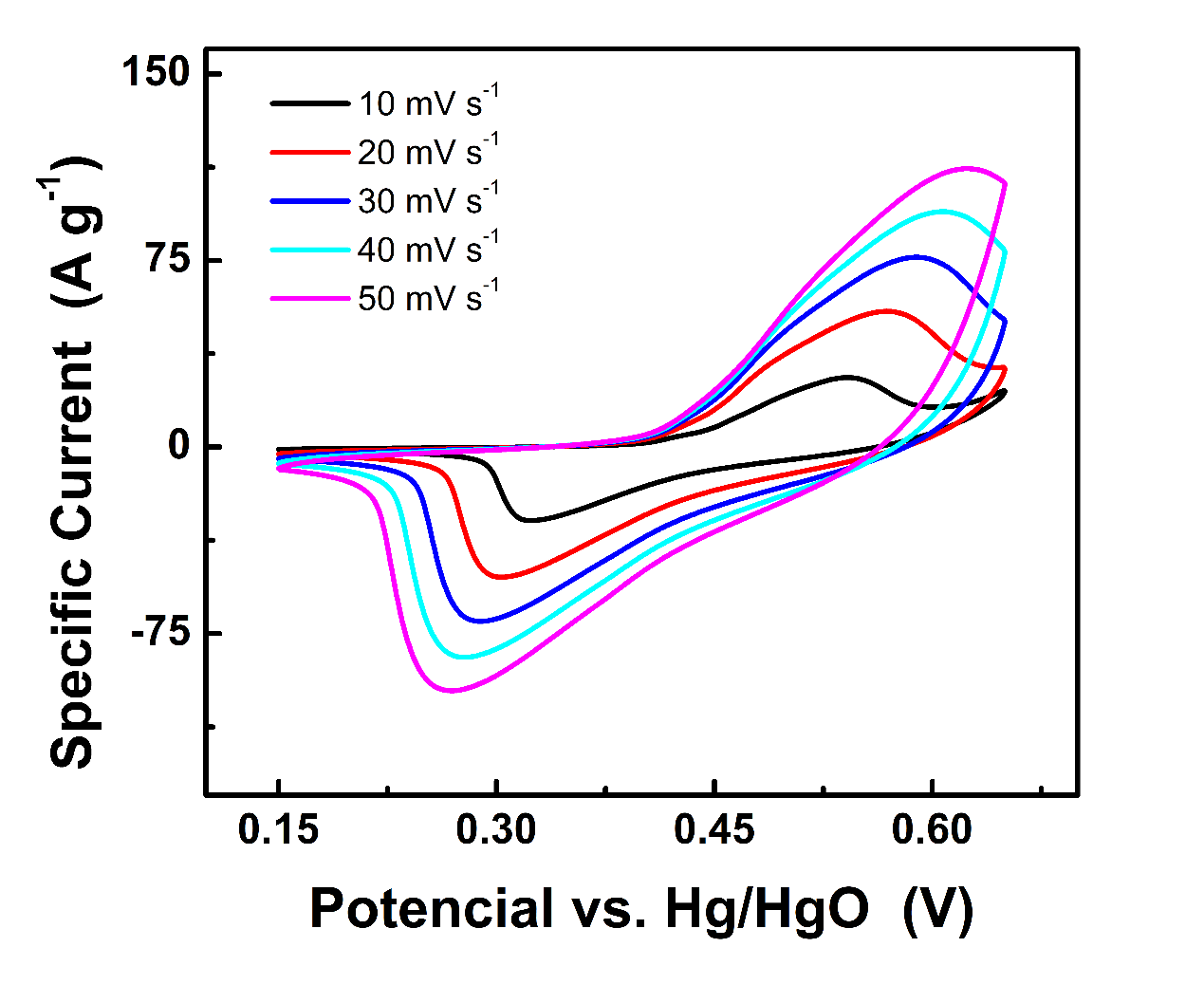


**Figure S1.** CV curves at scan rates of 10-50 mV s^-1^ of LONH at voltage window of 0.15-0.65 V in 3.0 M KOH.





**Figure S2.** GCD curves of LONH with current densities of 1.0-5.0 A g^-1^.





**Figure S3**. line chart derived from the discharging curves at different current densities of 1.0-5.0 A g^-1^ of LONH





**Figure S4.** The GCD curves of LONH and prisine Ni(OH)_2_ at 1.0 A g^-1^.


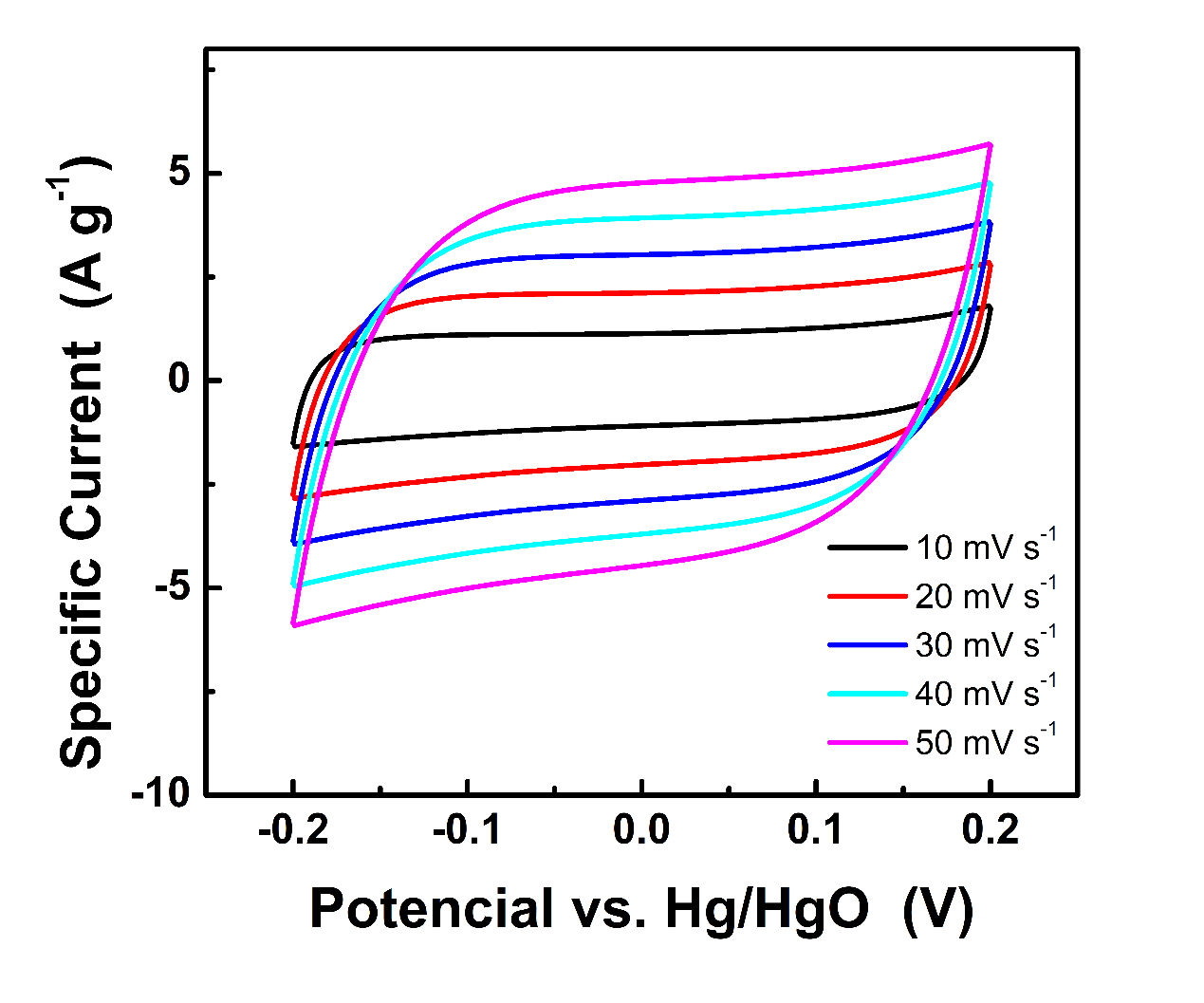


**Figure S5.** CV curves at scan rates of 10-50 mV s^-1^ of AC at voltage window of -0.2-0.2 V.


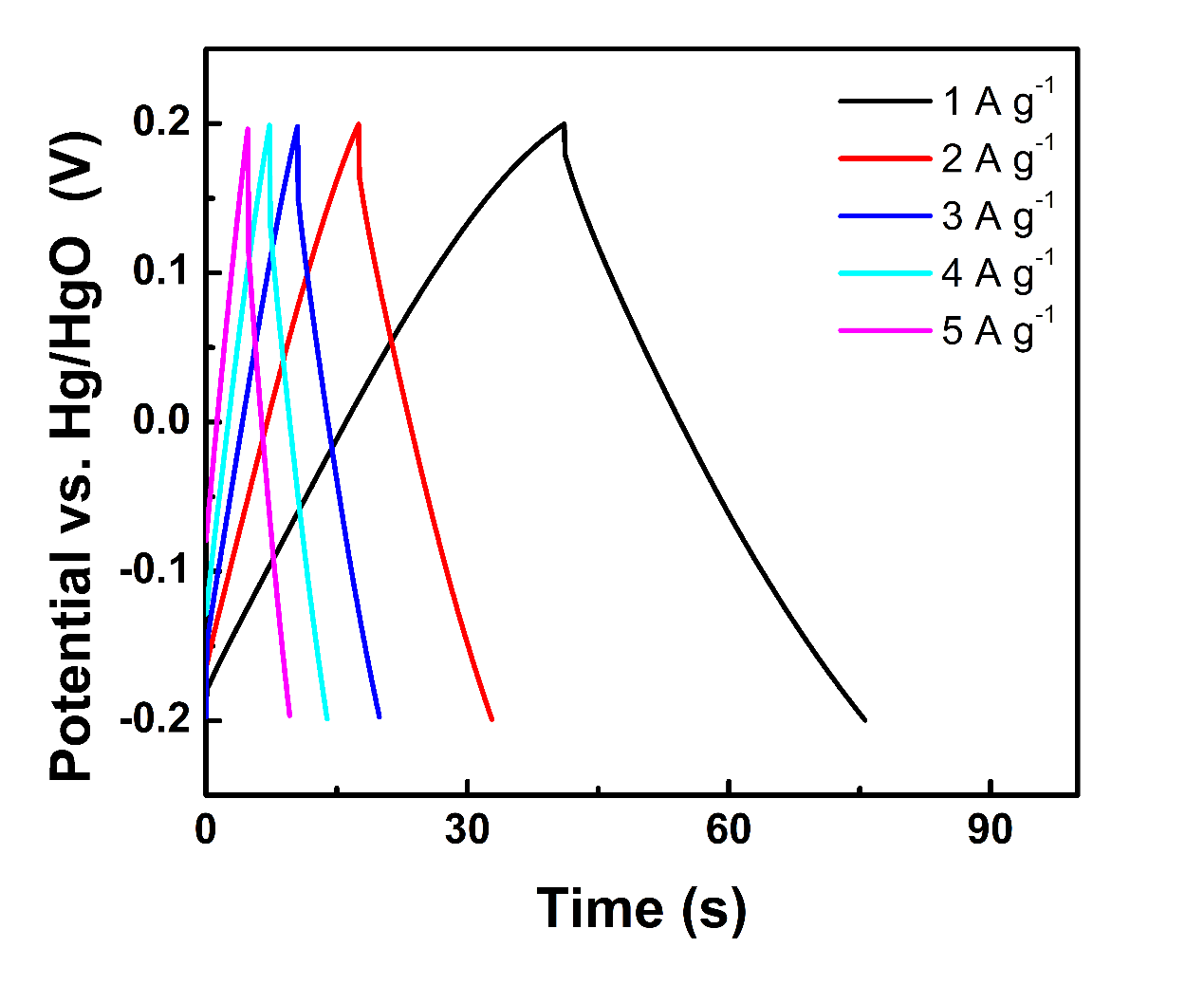


**Figure S6.** GCD curves at scan rates of 10-50 mV s^-1^ of AC at voltage window of -0.2-0.2 V.


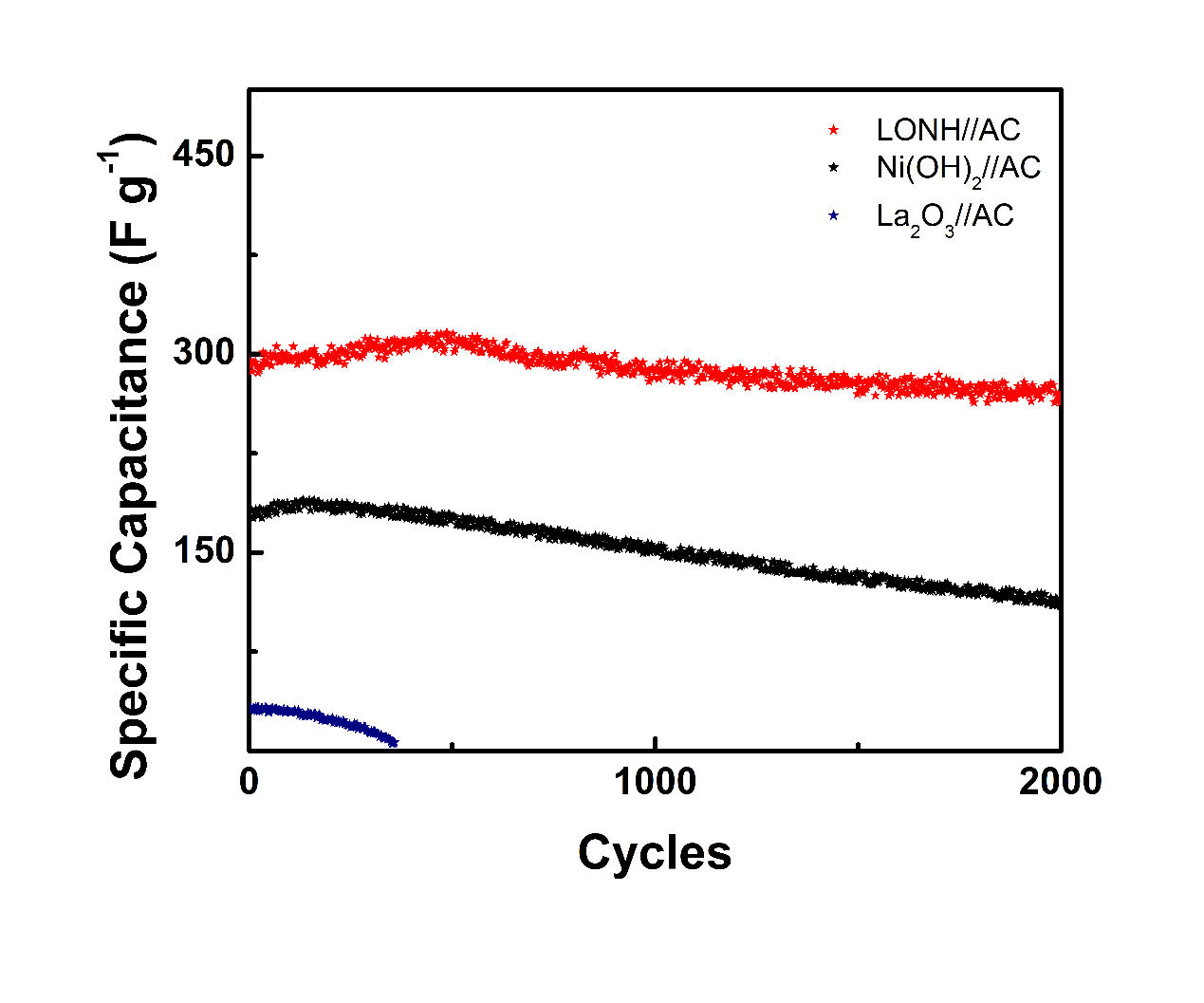


**Figure S7.** Specific capacitances of LONH//AC, Ni(OH)_2_//AC ,and La_2_O_3_//AC during charge-discharge cycling test at 2.0 A g^-1^.

**Table S2.** Comparison of the some as-reported asymmetric supercapacitors.

|  | Energy density  (Wh kg^-1^) | Power density (W kg^-1^) | Ref. |
| --- | --- | --- | --- |
| LONH | 107.8 | 800 | This work |
| PANI/La-10 | 56.1 | 400 | Morshed et al., 2021 |
| Ce-MOF-0.5 | 31.1 | 800 | Rabani et al., 2021 |
| NiV-LDH (2:2)//Bi_2_O_3_ | 65.5 | 1952.2 | Das et al., 2021 |
| MOF-Ce | 40.0 | 180 | Ghosh et al., 2019 |
| PrO_x_/CNT//V_2_O_5_/graphene | 52.1 | 2900 | Paravannoor et al., 2020 |
| La-Ni(OH)_2_/MWCNT | 25 | 1000 | Chakrabarty and Chakraborty, 2019 |
